# Comparative performance of the BGISEQ-500 and Illumina HiSeq4000 sequencing platforms for transcriptome analysis in plants

**DOI:** 10.1186/s13007-018-0337-0

**Published:** 2018-08-13

**Authors:** Fu-Yuan Zhu, Mo-Xian Chen, Neng-Hui Ye, Wang-Min Qiao, Bei Gao, Wai-Ki Law, Yuan Tian, Dong Zhang, Di Zhang, Tie-Yuan Liu, Qi-Juan Hu, Yun-Ying Cao, Ze-Zhuo Su, Jianhua Zhang, Ying-Gao Liu

**Affiliations:** 1 State Key Laboratory of Crop Biology, College of Life Science, Shandong Agricultural University, Taian, Shandong China; 2 Co-Innovation Center for Sustainable Forestry in Southern China, College of Biology and the Environment, Nanjing Forestry University, Nanjing, 210037 Jiangsu Province China; 3 Shenzhen Research Institute, The Chinese University of Hong Kong, Shenzhen, China; 4 School of Life Sciences, The Chinese University of Hong Kong, Shatin, Hong Kong; 5 Southern Regional Collaborative Innovation Center for Grain and Oil Crops in China, Hunan Agricultural University, Changsha, 410128 China; 6 BGI-Shenzhen, Shenzhen, People’s Republic of China; 7 College of Life Sciences, Nantong University, Nantong, Jiangsu China; 8 Department of Orthopaedics and Traumatology, The University of Hong Kong, Hong Kong, Hong Kong, SAR; 9 Department of Biology, Hong Kong Baptist University, and State Key Laboratory of Agrobiotechnology, The Chinese University of Hong Kong, Shatin, Hong Kong

**Keywords:** Alternative splicing, BGISEQ-500, Differential expressed genes, Illumina HiSeq4000, Next-generation sequencing, Transcriptome

## Abstract

**Background:**

The next-generation sequencing (NGS) technology has greatly facilitated genomic and transcriptomic studies, contributing significantly in expanding the current knowledge on genome and transcriptome. However, the continually evolving variety of sequencing platforms, protocols and analytical pipelines has led the research community to focus on cross-platform evaluation and standardization. As a NGS pioneer in China, the Beijing Genomics Institute (BGI) has announced its own NGS platform designated as BGISEQ-500, since 2016. The capability of this platform in large-scale DNA sequencing and small RNA analysis has been already evaluated. However, the comparative performance of BGISEQ-500 platform in transcriptome analysis remains yet to be elucidated. The Illumina series, a leading sequencing platform in China’s sequencing market, would be a preferable reference to evaluate new platforms.

**Methods:**

To this end, we describe a cross-platform comparative study between BGISEQ-500 and Illumina HiSeq4000 for analysis of *Arabidopsis thaliana* WT (Col 0) transcriptome. The key parameters in RNA sequencing and transcriptomic data processing were assessed in biological replicate experiments, using aforesaid platforms.

**Results:**

The results from the two platforms BGISEQ-500 and Illumina HiSeq4000 shared high concordance in both inter- (correlation, 0.88–0.93) and intra-platform (correlation, 0.95–0.98) comparison for gene quantification, identification of differentially expressed genes and alternative splicing events. However, the two platforms yielded highly variable interpretation results for single nucleotide polymorphism and insertion–deletion analysis.

**Conclusion:**

The present case study provides a comprehensive reference dataset to validate the capability of BGISEQ-500 enabling it to be established as a competitive and reliable platform in plant transcriptome analysis.

**Electronic supplementary material:**

The online version of this article (10.1186/s13007-018-0337-0) contains supplementary material, which is available to authorized users.

## Background

The past three decades witnessed a rapid advance in functional genomics, where gene transcription has emerged as an important research indicator for the study of functional genomics. Recently, transcriptome analysis has been accepted as a popular large profiling technique to reveal gene regulatory networks in both animals and plants [1–3]. The collection of methods which comprehensively and systematically analyze the transcriptome has been steadily increasing, both in their throughput and application range, eventually leading to better quality of data. Initial attempts regarding transcriptome analysis are based on oligonucleotide hybridization and array technologies. The establishment and gradual progression of next generation sequencing devices has resulted in high throughput RNA sequencing (RNA-seq) technology, defined as next-generation sequencing (NGS) [1–3], a routine laboratory practice in transcriptome analysis. The NGS’s capability of profiling the entire transcriptome, in addition to whole genomes, exomes and targeted gene regions and its dynamic range to detect subtle changes in expression level, has made significant impact in academic research, diagnostics and industry [1–3]. Since the last decade, the majority of efforts focused on reducing the prime cost while increasing sequencing accuracy and throughput for NGS platforms. Different from array-based technology, RNA-seq expands our knowledge on pervasive transcription of eukaryotic transcriptomes [4, 5], which enables to uncover the unexpected complexity of genomic regions which were once considered silent or antisense genes. The NGS approach has facilitated convenient and detailed study of new and informative features of transcription, such as novel transcript assembly, the regulation of untranslated regions (UTR), alternative splicing variants and the generation of small or non-coding RNAs [6–9]. Furthermore, during recent years, the comparison between RNA-seq and microarrays for transcriptome analysis has been carried out by several research groups. Particularly, the superior performance of RNA-seq is mainly attributed to its better resolution, lower variation and higher dynamic range than microarray-based transcriptome analysis [10–13]. However, the potential and capacity of RNA-seq needs to be explored in depth, and should be carefully investigated based on case studies and appropriate bioinformatic tools.

At present, a number of sequencing platforms such as Illumina HiSeq series and Roche 454 platform and RS/SEQUEL series from Pacific Biosciences (PacBio) have been developed by leading sequencing service providers worldwide. However, each platform is differed in its instrumentation and sequencing protocols such as library preparation procedures, base-calling mechanisms and measurement technology [14–17]. Thus, comparative studies among different sequencing platforms have been conducted to assess the intra- and inter-platform repeatability and reproducibility by using targeted RNA samples [18–20]. One classic example is from a case study in model yeast *Saccharomyces cerevisiae* [18], which focused on detailed intra-platform comparison adopting Illumina HiSeq series. The above study assessed the robustness of different platforms in of gene quantification, three different alignment algorithms, two assembly strategies (reference genome-based and de novo assembly) and five statistical methods have been used in order to validate the consistency of identifying differentially expressed gene (DEG) within Illumina HiSeq platforms and in comparison to conventional microarray datasets. High correlation has been reported between Illumina HiSeq and array based analysis using different combination of aforementioned approaches. Another large-scale cross-platform comparison has been performed by the Association of Biomolecular Resource Facilities (ABRF) members [19]. In total, replicate experiments from 15 laboratories, 4 library construction protocols, 3 size fractions of library and 5 sequencing platforms have been subjected for comprehensive evaluation. The outcome of this cross-site comparison study among 15 ABRF laboratories has generated an early standard for performing NGS analysis on animal samples.

Although single molecule long-read sequencing platform has been developed since several years ago, representative platforms such as PacBio series and Minion (Oxford Nanopore Technologies) are not widely used in plant transcriptome studies accounting to their high cost and low throughput [21]. The above two platforms have yet to reach the performance and dynamic range similar to those of NGS platforms with further advancement of their sequencing technology. At present, a wide range of NGS platforms are available in the sequencing market of China. Among these platforms, the Illumina series had gained its position as one of the most widely used sequencing platforms and have generated a substantial part of transcriptome data in the past 5 years probably due to its stable performance, lower error rate and relatively low cost in transcriptome analysis. In 2016, BGI announced its own NGS platform designated as BGISEQ-500 [22]. The general NGS workflow and stepwise sequencing procedures of the newly developed BGISEQ-500 are similar to those of Illumina series; yet the two templates have marked differences. The subsequent DNA nanoball technology specifically used for library preparation in BGISEQ-500 platform is different from the library construction protocol used in Illumina series [23]. Initial tests in DNA sequencing confirmed the platform’s potential to generate high quality data in DNA-related NGS applications [24–26]. The performance of this platform has been subsequently validated in small RNA profiling in comparison to Illumina series [20]. BGISEQ-500 utilizes both single end (SE) and paired end (PE) modes which are comparable to latest Illumina model, HiSeq4000. The announced data on throughput of BGISEQ-500 was relatively high, which is potentially suitable for high throughput transcriptome studies. Even though it has been already validated for small RNA profiling, a comprehensive evaluation of the performance of BGISEQ-500 in transcriptome analysis has not been recorded to date. To this end, we used two *Arabidopsis thaliana* (WT, Col-0) seedling samples exposed to dimethyl sulfoxide (DMSO) or abscisic acid (ABA) (three replicates of each), to explore the capability of this platform in transcriptomic profiling. For comparison, Illumina HiSeq4000 was used as a control platform along with BGISEQ-500. Key parameters in current plant transcriptome studies including DEGs, alternatively spliced (AS) events, single nucleotide polymorphism (SNP), and insertions–deletions (INDEL) were comprehensively validated between these two platforms. Results indicated that both platforms have high inter- and intra-platform repeatability in gene quantification, DEG and AS analysis, but present a relative low correlation in SNP and INDEL identification. We discuss the possible underlying causes for the above and put forward our suggestions for enhancement of transcriptome analysis with respect to the two platforms subjected for comparison.

## Results

### RNA samples and sequencing protocols for inter-platform comparison

**Fig. 1:**
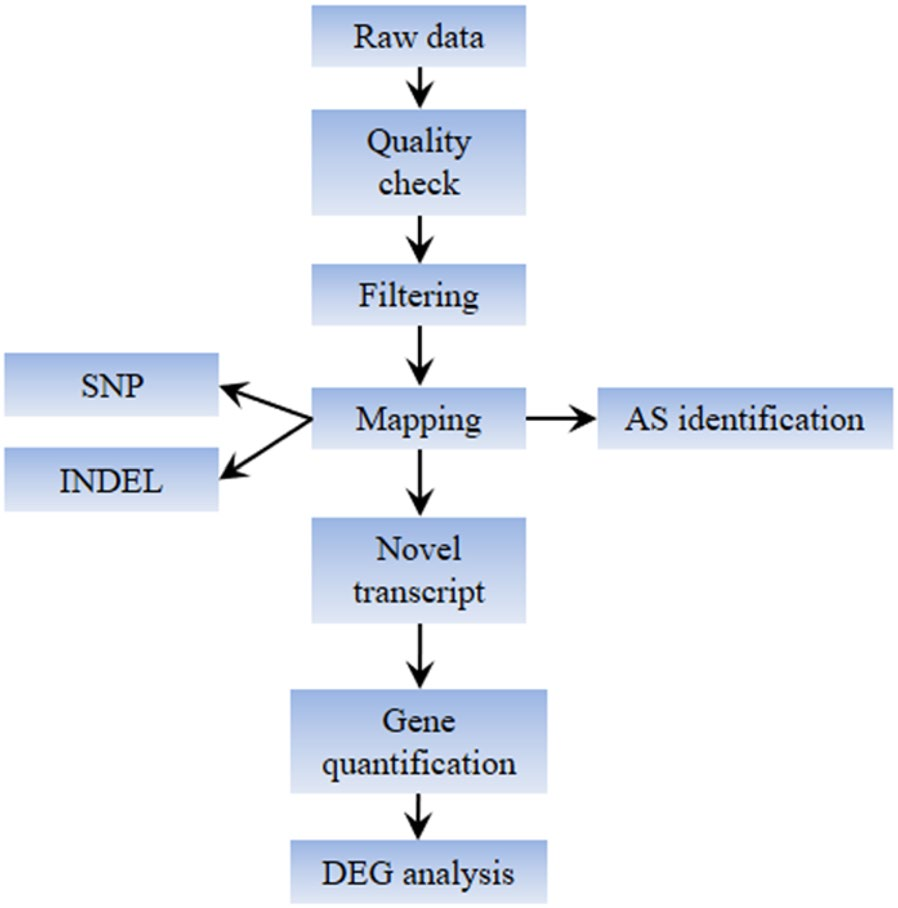
Schematic view of analytical pipeline of this study. *SNP* single nucleotide polymorphism, *INDEL* insertion–deletion, *AS* alternative splicing, *DEG* differentially expressed genes

Being an in-house developed sequencing platform, the capability of BGISEQ-500 in transcriptome analysis needed to be verified using a range of target biological samples along with a reference platform as a standard. Meanwhile, Illumina HiSeq4000 is currently recognized as a widely used sequencing platform and gained popularity in the sequencing market of China, and thereby proves to be a suitable reference platform in evaluation of newly developed platforms. Therefore, we performed a comparison between the Illumina HiSeq4000 and BGISEQ-500 in two NGS applications; transcriptomic profiling and identification of alternative splicing. Two RNA samples, DMSO and ABA-treated *Arabidopsis* seedlings (triplicate per sample), were used for this comparison. The functionality of the two platforms of our consideration differs from each other in several aspects. Accordingly, the library construction protocol for BGISEQ-500 sequencing was different from that of for HiSeq platforms. The procedure of bubble adapter ligation in BGISEQ series library preparations is a unique step and is patent protected. Furthermore, incorporation of DNA nanoball (DNB) technology during the library construction steps in BGISEQ-500 has yielded several benefits. Initially, the formation of DNA nanoball is based on the rolling-circle replication which utilizes the same original template circle to generate each new copy, ensuring that replication errors are minimized and prevented from amplification. Secondly, millions of nanospots which contain more DNA copies guarantee a high SNR imaging for accurate and precise base calling. Thirdly, cPAS chemistry, along with linear RCR amplification allows higher sensitivity for identification of low-abundance/expressed species with high call confidence. Last but not the least, single-tube library preparation, carried out in a single low-volume solution, allows easy process automation for more consistent results. An overview of this protocol is presented in supplemental materials of this article (Additional file 1: Figure S1). Initially, we applied strand-specific libraries for PE100 sequencing mode in both platforms. The sequencing mode PE75 from strand-nonspecific libraries was used as an additional dataset for comparison. The brief analytical pipeline for both platforms including initial quality check, reads filtering, mapping to *Arabidopsis* genome and subsequent transcriptome-related analysis following the standard procedures has been published previously (Fig. 1) [27, 28]. The quality assessment and platform comparison will be discussed in the forthcoming sections.

### Base and raw data quality

**Fig. 2:**
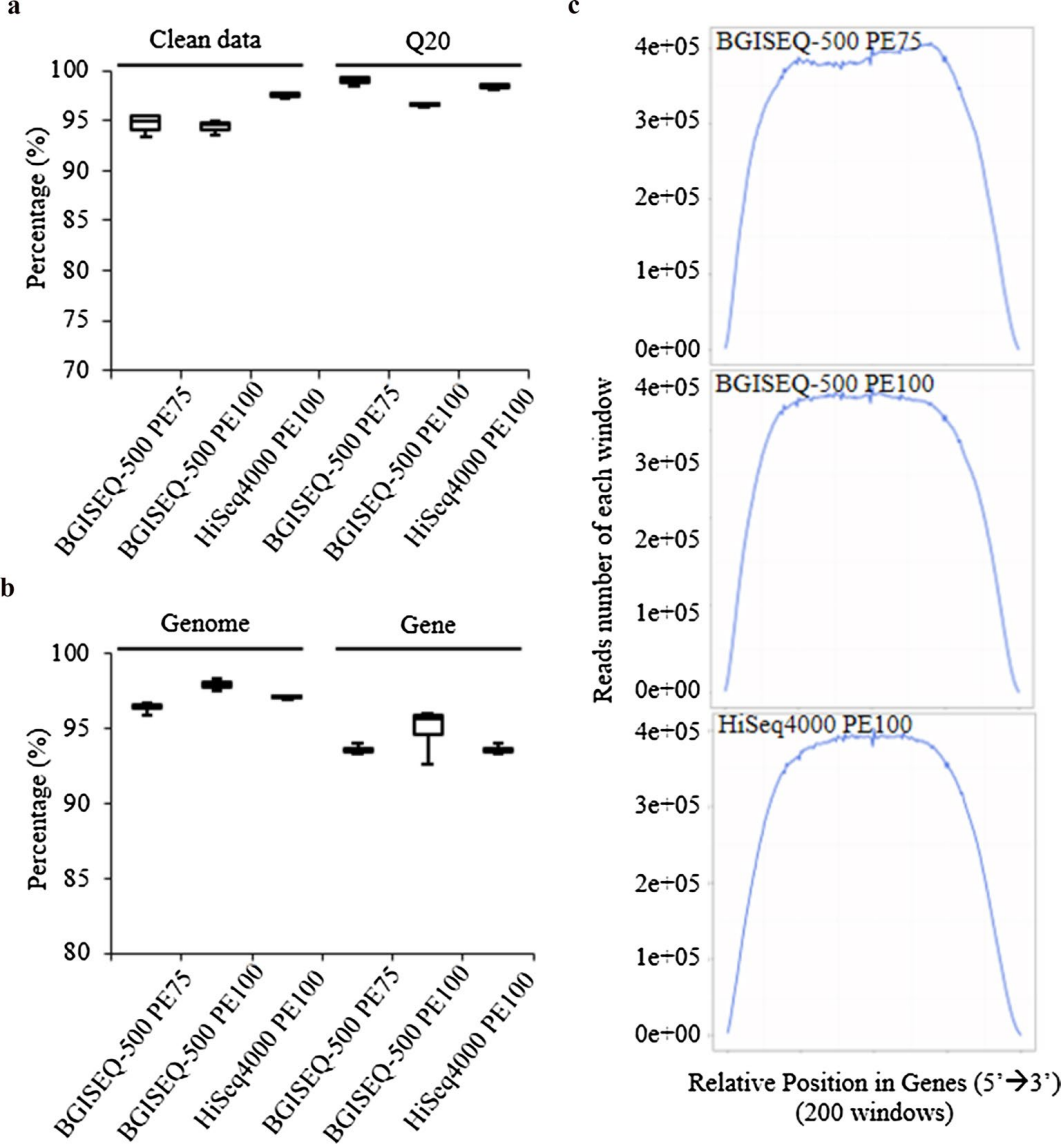
Comparison of sequencing quality among BGISEQ-500 PE75, BGISEQ-500 PE100 and HiSeq4000 PE100. **a** Base quality representation for clean reads and Q20. **b** Reads quality evaluation and mapping percentage. **c** Reads distribution along the relative position of genes

The basic parameters of three sequencing datasets are presented for all the replicates (Table 1). Approximately an average of 70 Mb raw reads was produced for each replicate. Among these, over 94% of data were regarded as clean data after filtering. The percentage of clean reads was comparable between HiSeq4000 PE100 and BGISEQ-500 PE100, whereas the average percentage of clean reads in BGISEQ-500 PE75 samples was higher than those in the other two datasets (Table 1 and Fig. 2a). In addition, quality values were recorded for all the replicates and Q20 comparison showed that BGISEQ-500 PE75 took advantage than the other two sequencing modes (Fig. 2a) data quality. In particular, all three platforms were detected with biased quality values in first 16 bases, which is a generally known effect caused by reverse transcriptase at priming step during library preparation [29]. This phenomenon also affected the GC composition among all the replicates (Additional file 1: Figure S2). Subsequent reads mapping was performed among reads from three sequencing datasets. On average, over 96% and 91% of clean reads could be mapped to *Arabidopsis* genome and genes for all the three datasets, respectively. No obvious differences were observed among these three datasets (Fig. 2b). However, two BGISEQ datasets, BGISEQ-500 PE100 and BGISEQ-500 PE75 on average, possessed a slightly higher percentage of mapped reads in comparison with the data generated by HiSeq4000 PE100 mode. Furthermore, no variations were detected among the three sequencing methods in reads distribution along genes (Fig. 2c), suggesting that BGISEQ-500 platform can achieve similar sequencing quality to HiSeq4000 using both PE and SE modes for transcriptome analysis.

**Table 1:** Summary of basic parameters in three RNA sequencing datasets

	Sample	Total raw reads (Mb)	Total clean reads (Mb)	Genome mapped reads (Mb)	Gene mapped reads (Mb)	Genome mapping rate (%)	Gene mapping rate (%)
HI-SEQ4000 PE100	1_DMSO_6 h_1	70.14	65.81	63.59	60.22	96.62	91.50
	2_DMSO_6h_2	70.14	67.00	64.78	61.59	96.68	91.92
	3_DMSO_6h_3	70.14	65.54	63.09	59.47	96.26	90.74
	4_ABA_6h_1	70.14	66.44	63.52	59.48	95.60	89.53
	5_ABA_6h_2	70.14	67.02	64.72	61.60	96.57	91.92
	6_ABA_6h_3	70.14	66.86	64.46	60.82	96.41	90.97
BGI-SEQ500 PE100	1_DMSO_6h_1	72.10	67.00	65.44	61.19	97.67	91.33
	2_DMSO_6h_2	69.63	65.85	64.53	63.06	97.99	95.77
	3_DMSO_6hJ_	69.62	65.80	64.14	62.02	97.47	94.25
	4_ABA_6h_1	69.57	65.99	64.69	62.97	98.03	95.42
	5_ABA_6h_2	69.57	65.78	64.63	63.13	98.25	95.97
	6_ABA_6h_3	69.64	65.47	64.16	62.75	98.00	95.85
BGI-SEQ500 PE75	1_DMSO_6h_1	69.50	67.80	65.76	63.39	96.99	93.50
	2_DMSO_6h_2	69.69	67.79	65.49	63.25	96.60	93.31
	3_DMSO_6h_3	68.16	66.41	64.41	61.99	96.99	93.34
	4_ABA_6h_1	67.36	65.67	63.71	61.49	97.02	93.64
	5_ABA_6h_2	67.92	66.26	64.25	62.05	96.96	93.64
	6_ABA_6h_3	69.71	68.02	66.68	64.06	98.03	94.18

### Intra- and inter-platform comparison of gene detection and quantification

**Fig. 3:**
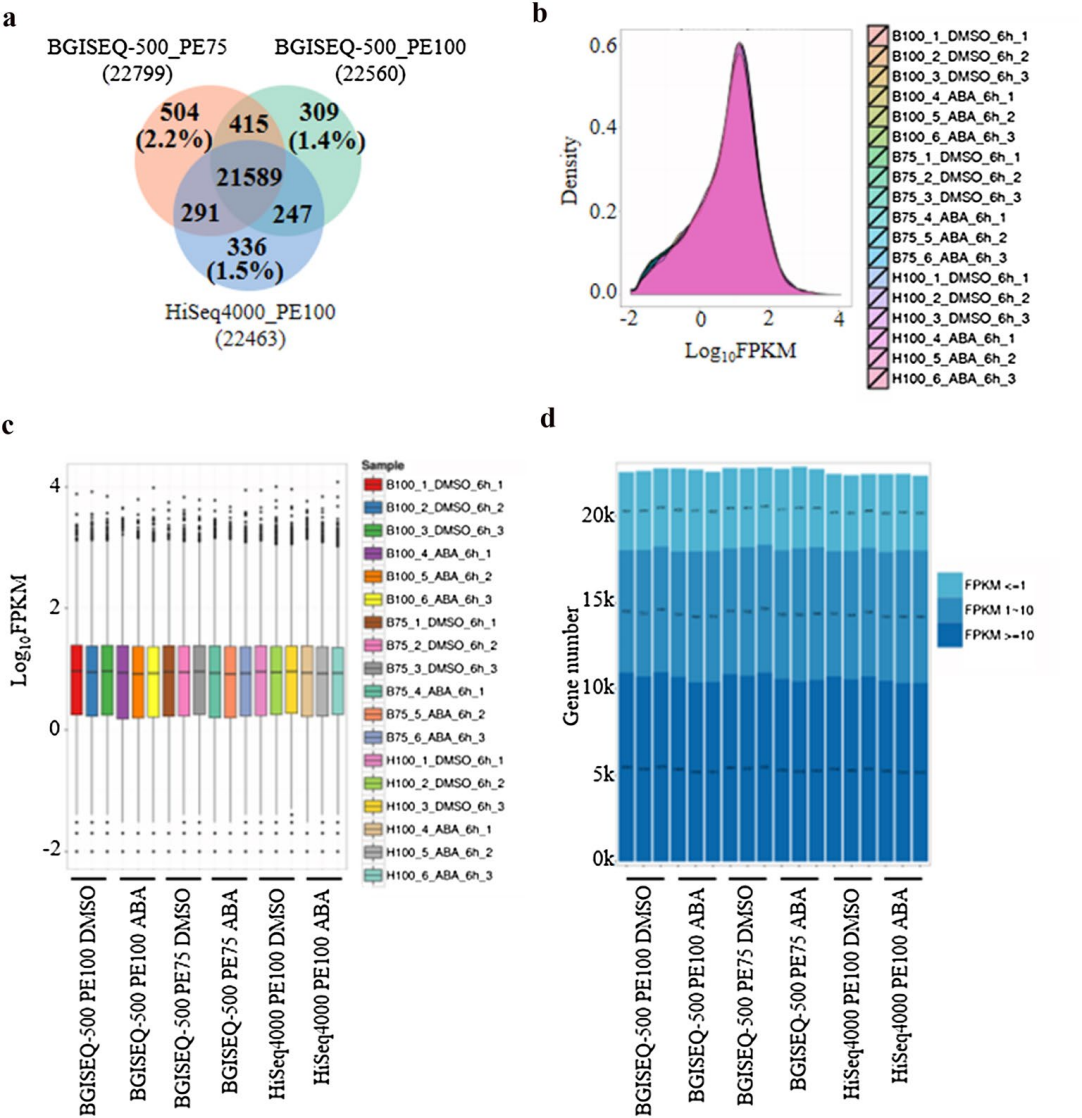
Repeatability of gene detection and quantification among three sequencing approaches. **a** Venn diagram representation of gene detection. Expression density distribution (**b**), boxplot gene expression graph (**c**), high and low abundance transcripts quantification (**d**) for all the replicates tested by three sequencing approaches in this study

In transcriptome analysis, the identification of DEG is of considerable importance for the majority of research projects. Thus, we further compared the capacity of BGISEQ-500 and HiSeq4000 platforms on gene detection and quantification. Approximately 22,000 genes were detected across the three datasets. Over 97% of genes were commonly detected by all three sequencing approaches (Fig. 3a). In addition, the two BGISEQ approaches were slightly higher in their gene detection and quantification with respect to the number of total identified genes and transcripts, but lower in the number of identified novel genes and transcripts than that of HiSeq approach (Additional file 2: Table S1). All three approaches shared fairly consistent expression density distribution and accuracy in quantification of both low and high abundance genes (Fig. 3b–d). Intriguingly, hundreds of method-specific genes were identified uniquely by each sequencing approach (Fig. 3a). In comparison to unique genes identified by HiSeq4000, most of the unique genes detected by BGISEQ-500 platform were concentrated at low abundance interval (*i.e.* FPKM ≤ 1) (Additional file 1: Figure S3A–C). We suggest that the detection of a greater number of low abundance transcripts by BGISEQ-500 could be a consequence of the nanoball-based linear amplification feature utilized by the platform. Furthermore, the intra- and inter-platform repeatability was assessed by using one sample named as DMSO_6h_1. All three sequencing methods showed a high level of Spearman and Pearson correlations when two parallel libraries generated from the same sample were used in analysis (Additional file 1: Figure S4A, B). Similarly, inter-platform measurement showed high consistency in gene quantification among all three sequencing approaches with minor differences. In particular, both Spearman and Pearson correlation rankings for the 3 sequencing approaches in their gene detection and quantification are as follows: HiSeq4000 PE100 versus BGISEQ-500 PE100 > BGISEQ-500 PE75 versus BGISEQ-500 PE100 > HiSeq4000 PE100 versus BGISEQ-500 PE75 (Additional file 1: Figure S4C, D).

### Detection of differentially expressed genes and alternative splicing

**Fig. 4:**
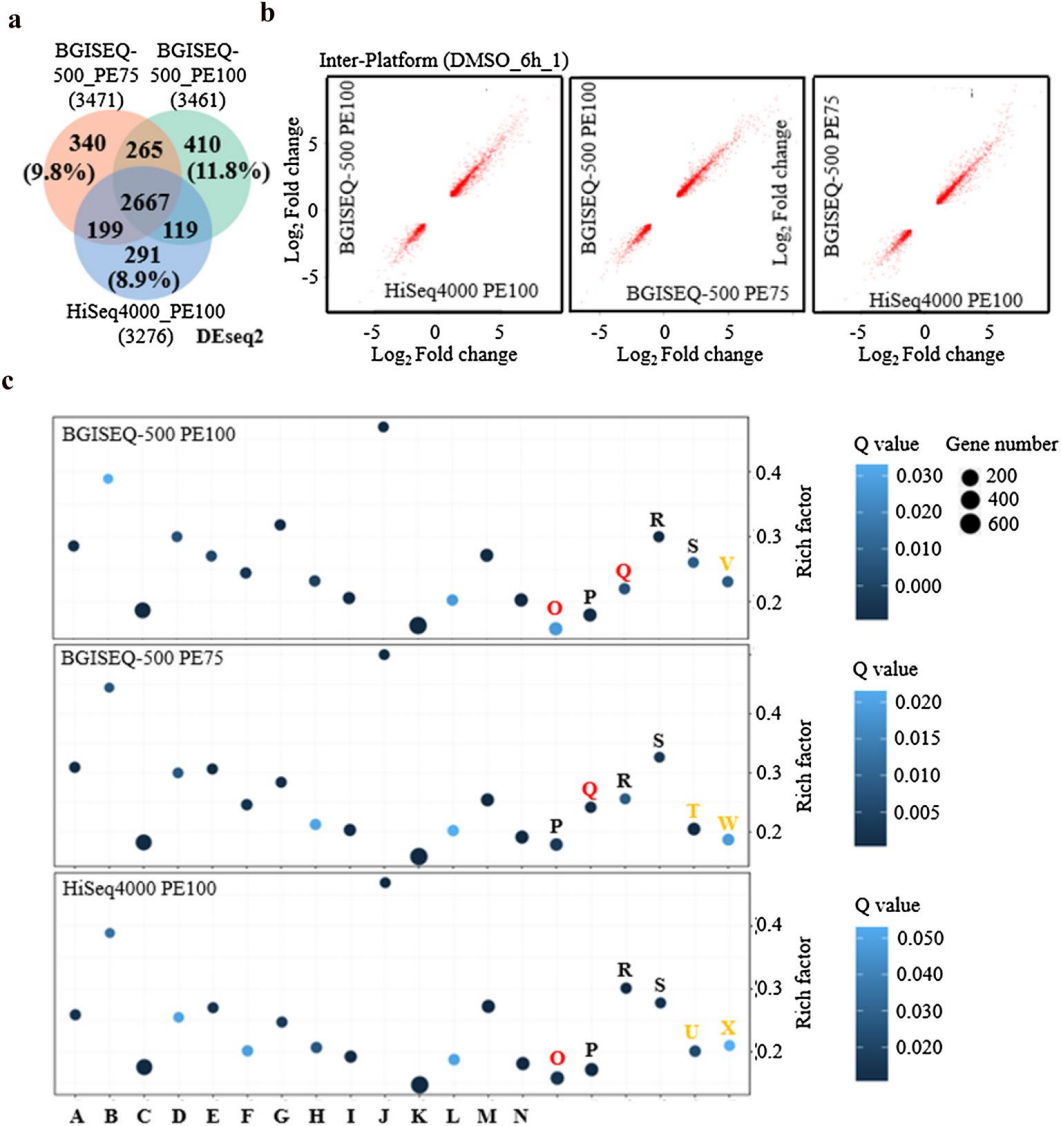
Differentially expressed genes determination among three sequencing approaches. **a** Venn diagram representation of DEG calling in each sequencing approach. **b** Cross-platform comparison in DEG detection. **c** Pathway enrichment of each sequencing approach. Black, pathways enriched in all the three approaches; Red, pathways enriched in two approaches; Orange, pathways enriched in one approach. A, alpha-Linolenic acid metabolism; B, Anthocyanin biosynthesis; C, Biosynthesis of secondary metabolites; D, Biosynthesis of unsaturated fatty acids; E, Carotenoid biosynthesis; F, Cutin, suberine and wax biosynthesis; G, Flavonoid biosynthesis; H, Galactose metabolism; I, Glycerolipid metabolism; J, Indole alkaloid biosynthesis; K, Metabolic pathways; L, Other glycan degradation; M, Biosynthesis of secondary metabolites in phenylpropanoid pathway; N, Plant hormone signal transduction; O, Plant-pathogen interaction; P, Starch and sucrose metabolism; Q, Phenylpropanoid biosynthesis; R, Other terpenoid biosynthesis; S, Zeatin biosynthesis; T, MAPK signaling pathway; U, Peroxisome; V, Fatty acid metabolism; W, Pentose and glucuronate interconversions; X, Phenylalanine metabolism

To examine the ability of BGISEQ-500 and Illumina platforms in DEG detection, we compared the DEG lists identified from above three sequencing approaches. The analytical pipeline was normalized for all repeats and significant DEGs were defined as FDR ≤ 0.05 and fold change ≥ 2. In total, over 3200 DEGs were identified in each sequencing approach by DEseq2, a well-known method for DEG identification. Both BGISEQ-500 PE100 and BGISEQ-500 PE75 were able to identify approximately 200 more DEGs than that of HiSeq4000 PE100 mode. All the three approaches detected a higher percentage of DEGs (~ 90%) (Fig. 4a), indicating high correlations among three sequencing methods in two different platforms (Fig. 4b). However, approximately 10% of DEGs were uniquely present in each sequencing approach (Fig. 4a), implying approach-specificity of each sequencing method. Furthermore, pathway and gene ontology (GO) analysis revealed that the three sequencing approaches could result in similar biological interpretations (Fig. 4c and Additional file 1: Figure S5). Out of the topmost 20 biological pathways identified during analysis, seventeen pathways were commonly detected by all the three sequencing approaches (Fig. 4c), and a similar number of GO terms was enriched as well (Additional file 1: Figure S5). In order to test the validity of biological significance presented by the above three sequencing approaches, four additional methods were then applied for DEG identification, namely; AudicS, Cuffdiff, DEGseq and edgeR (Additional file 1: Figure S6A–D). As described previously, different DEG calling methods may result in varying total number of DEGs. However, a high concordance of biological interpretation was detected among all the DEG datasets generated by these five different methods (Additional file 1: Figures S6E, S7–10).

**Fig. 5:**
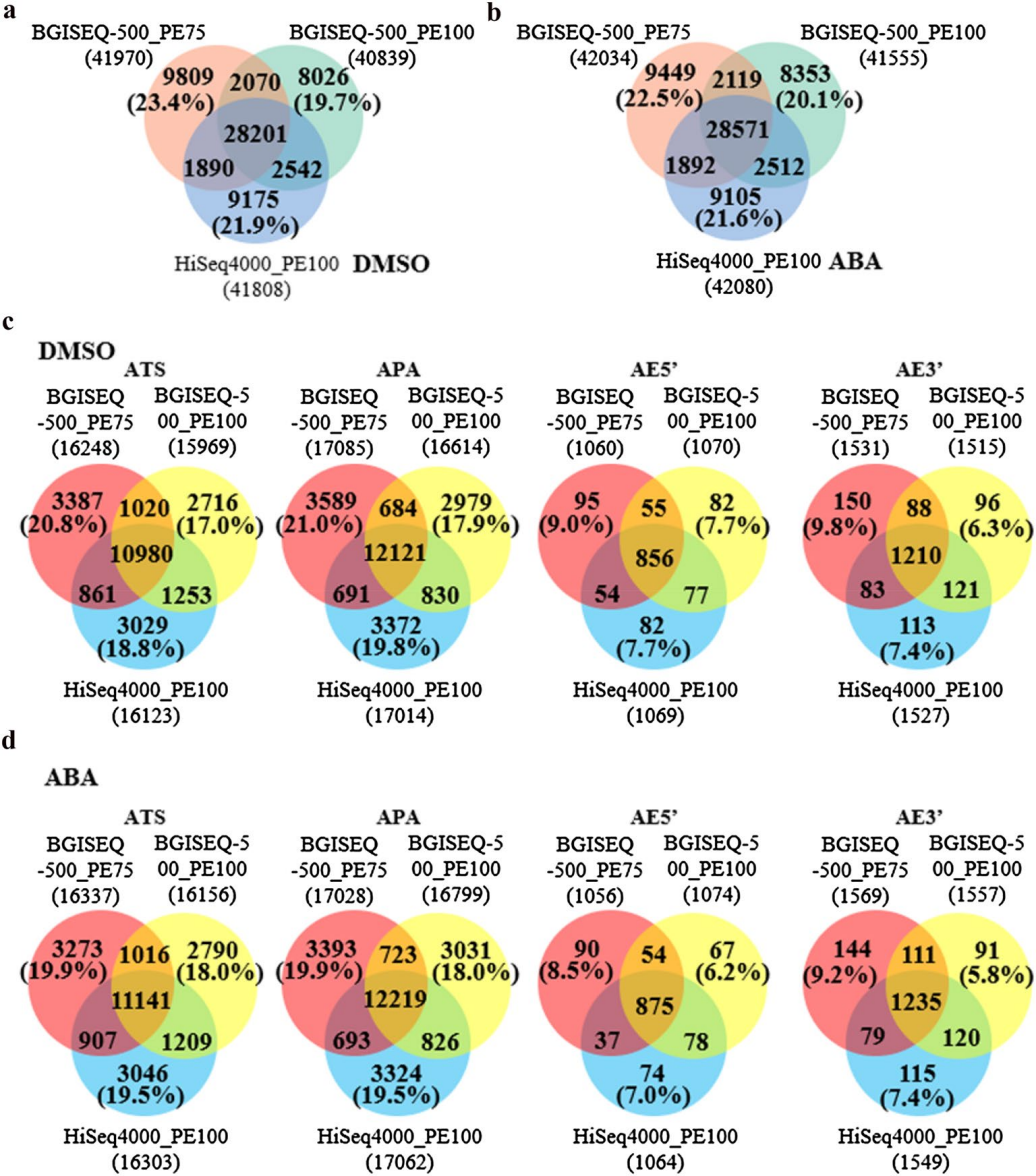
Comparison of alternative spliced events identification. Venn diagrams representation of AS events identification in DMSO- (**a**) and ABA-treated (**b**) samples by each sequencing approach. Venn diagrams to represent **c** AS events in DMSO-treated and **d** ABA-treated samples. *ATS* alternative transcription start, *APA* alternative polyadenylation, *AE5′* alternative 5′ splice site, *AE3′* alternative 3′ splice site

As an important post-transcriptional modification, alternative splicing (AS) is gaining much attention in recent years, as one of the major mechanisms to generate transcriptome diversity. In this context, the ability of each sequencing platform under comparison to detect splicing junction and corresponding alternative splicing pattern were subsequently analysed across transcriptomes. In general, all three datasets detected over 40,000 AS events in both DMSO- and ABA-treated samples (Fig. 5). In contrast to the above level of detection, the percentage of AS events commonly to the three datasets was less than 80%. In comparison to the outcome of DEGs and gene quantification by the 3 sequencing approaches, the percentage of common AS events in the datasets generated by the above methods show a larger variation. Among these, two post-transcriptional events (alternative transcription start, ATS and alternative polyadenylation, APA), which has been reported to affect the transcript diversity [30], had similar variation range in comparison to that of total AS events (Fig. 5c, d). Particularly, alternative 5′ splice site (AE5′) and alternative 3′ splice site (AE3′) showed lower variation range (6–9%) among three sequencing approaches than the average variation range detected for the total AS events (19–24%) (Fig. 5c, d). In contrast, in the cases of exon skipping (SKIP), multiple exon skipping (MSKIP), intron-retention (IR) and multiple intron-retention, BGISEQ-500 PE100 presented a dataset completely different from the other two sequencing approaches in ABA-treated samples (Additional file 1: Figure S11A, B). In general, intra-platform variations among three sequencing approaches were smaller than that of inter-platform comparisons (Additional file 1: Figures S12–14). Furthermore, each sequencing approach produced a distinct dataset on the identification of two recently identified AS events [21], alternative first exon (AFE) and alternative last exon (ALE) in both inter- (Additional file 1: Figure S11A, B) and intra-platform comparisons (Additional file 1: Figure S15). However, the causes of these variations remain unclear and need to be further investigated.

### Identification of single nucleotide polymorphisms and insertion–deletion mutation

Single nucleotide polymorphisms (SNP) are insertion–deletion mutations (INDELs) which are crucial genomic features to reveal genetic variation. High throughput transcriptome analysis is capable in investigating how these DNA variations can be transcribed into RNA messengers to affect subsequent protein function. In this study, we examined the competency of BGISEQ-500 sequencing platform to detect SNP variations at the transcript level. On average, BGISEQ-500 approaches (PE100 and PE75) were able to identify more SNP events than that of HiSeq4000 approach (Table 2 and Additional file 1: Figure S16). However, all three sequencing methods had relatively low repeatability for both intra- and inter-platform comparisons by using either transcript or CDS sequences. Only 30–40% of SNP events could be repeatedly identified in replicates. The repeatability of intra-platform comparison with respect to SNP detection was ranked as BGISEQ-500 PE100 > HiSeq4000 PE100 > BGISEQ-500 PE75 (Additional file 1: Figure S16). Similar observations were recorded in INDEL analysis (Table 3 and Additional file 1: Figure S17). The low repeatability of these two analyses could be accountable to the presence of non-repeatable 6N random primer introduced mutations during sequencing library construction.

**Table 2:** Summary of SNP identification

	Sample	A–G	C–T	Transition	A–C	A–T	C–G	G–T	Transversion	Total
HI-SEQ4000 PE100	1_DMSO_6h_1	264	158	422	84	84	57	71	296	718
	2_DMSO_6h_2	284	186	470	67	74	52	47	240	710
	3_DMSO_6h_3	254	202	456	62	66	49	57	234	690
	4_ABA_6h_1	297	201	498	91	96	58	68	313	811
	5_ABA_6h_2	250	192	442	75	66	42	64	247	689
	6_ABA_6h 3	248	181	429	63	86	43	51	243	672
	Average	266	187	453	74	79	50	60	262	715
BGI-SEQ500 PE100	1_DMSO_6h_1	491	264	755	240	305	49	77	671	1426
	2_DMSO_6h_2	342	177	519	178	244	38	48	508	1027
	3_DMSO_6h_3	460	223	683	323	411	40	67	841	1524
	4_ABA_6h_1	348	217	565	178	256	46	70	550	1115
	5_ABA_6h_2	324	211	535	165	209	41	47	462	997
	6_ABA_6h_3	308	200	508	150	189	31	41	411	919
	Average	379	215	594	206	269	41	58	574	1168
BGI-SEQ500 PE75	1_DMSO_6h_1	384	191	575	295	432	60	128	915	1490
	2_DMSO_6h_2	381	223	604	239	378	64	120	801	1405
	3_DMSO_6h_3	394	191	585	251	362	59	126	798	1383
	4_ABA_6h_1	398	178	576	292	368	57	105	822	1398
	5_ABA_6h_2	349	182	531	210	303	48	90	651	1182
	6_ABA_6h_3	323	167	490	202	273	44	90	609	1099
	Average	372	189	560	248	353	55	110	766	1326

**Table 3:** Summary of INDEL identification

	Sample name	Total number	Up2k	Exon	Intron	Down2k	Intergenic
HI-SEQ4000 PE100	1_DMSO_6h_1	813	45	611	108	30	19
	2_DMSO_6h_2	774	53	590	79	30	22
	3_DMSO_6h_3	736	41	561	94	25	15
	4_ABA_6h_1	893	48	675	128	28	14
	5_ABA_6h_2	776	43	589	105	24	15
	6_ABA_6h_3	820	40	618	123	31	8
	Average	802	45	607	106	28	16
BGI-SEQ500 PE100	1_DMSO_6h_1	2438	65	1905	403	46	19
	2_DMSO_6h_2	2342	72	1879	329	41	21
	3_DMSO_6h_3	2395	71	1832	416	52	24
	4_ABA_6h_1	2382	61	1898	359	40	24
	5_ABA_6h_2	2128	61	1731	287	33	16
	6_ABA_6h_3	1999	61	1617	278	31	12
	Average	2281	65	1810	345	41	19
BGI-SEQ500 PE75	1_DMSO_6h_1	1834	65	1552	163	43	11
	2_DMSO_6h_2	1459	59	1258	84	38	20
	3_DMSO_6h_3	2130	87	1789	175	54	25
	4_ABA_6h_1	1561	55	1318	131	42	15
	5_ABA_6h_2	1288	46	1125	68	33	16
	6_ABA_6h_3	1252	51	1094	67	28	12
	Average	1587	61	1356	115	40	17

## Discussion

### High performance of BGISEQ-500 in transcriptome analysis

Up to date, this article represents the first cross-platform comparison to evaluate BGISEQ-500 in transcriptome analysis. The results from the present study provide reference datasets to examine key parameters utilized in the transcriptome analytical pipeline. The intra- and inter-platform correlations of gene quantification, DEG detection, AS identification, SNP and INDEL detection between BGISEQ-500 and Illumina HiSeq4000 sequencing platforms have been critically evaluated. Previous reports mentioned that BGISEQ-500 has similar throughput and turnaround time to that of HiSeq2500 platform in DNA sequencing of human genome [25]. Furthermore, more even read distribution than HiSeq data has been observed in previous miRNA analysis with respect to BGISEQ-500 platform [20]. Although the throughput was not tested in the present study, a similar number of starting reads (~ 70 Mb) was used to facilitate subsequent normalization and comparison. Likewise, both base/read quality and read distribution pattern of BGISEQ-500 was comparable to those of HiSeq4000 (Fig. 2 and Table 1). In addition, high correlations of gene detection and DEGs/AS identification (Figs. 3, 4, 5) suggest that BGISEQ-500 has the capability for efficient transcriptome analysis. Especially, the consistency in biological interpretation from DEGs was supported by five different DEG calling methods. By incorporating more methods in DEG analysis such as *limma* [31], may further increase the confidence of this conclusion. However, the data generated in this study was solely based on model dicot plant *Arabidopsis*, for which high quality and rigorously annotated genomic information is already available. Further evaluation is required to test the transcriptome compatibility of BGISEQ-500, especially with respect for those plant species with no reference genome sequences available, or other eukaryotic samples such as animals and fungi.

In comparison to Illumina series, BGISEQ-500 possesses fundamental differences in terms of library preparation and sequencing strategy [23]. The success of this platform provides yet another set of reliable sequencing approaches for any experiments dealing with NGS analysis including research conducted using either DNA or RNA sequencing techniques. However, further improvements such as sequencing quality and standardized protocols, strand-specific library construction and bias correction, are needed to improve the performance of BGISEQ series for it to be applied in the other NGS sequencing applications (*e.g.* epigenomic and metagenomic sequencing; LncRNA analysis).

### Platform-based variations in transcriptome analysis

Although previous studies proposed that comprehensive data mining projects can be applied to datasets generated by different platforms despite the intrinsic variations [19], in our present work, we express our particular concern on the phenomena of platform variations and their effect in identification of AS, SNP and INDEL during transcriptome analysis. Previous comparative studies have indicated that considerable variation can be detected between BGISEQ-500 and HiSeq platforms in miRNA identification [20]. In our study, approximately 20% of AS was uniquely identified by each platform (Fig. 5), especially for AS events like AFE and ALE, which is shown large variations in both inter- and intra-platform comparisons (Additional file 1: Figures S11 and 15). Thus, the authenticity of such AS events is needed to be verified by parallel independent methods such as quantitative real-time PCR or RT-PCR. In comparison to the other two sequencing approaches, BGISEQ500-PE100 mode showed distinct AS identification in SKIP and IR-related events (Additional file 1: Figure S11B). However, the underlying causes for this variation remain to be investigated. We observed larger variations with respect to SNP and INDEL calling from our dataset (Additional file 1: Figures S16 and 17). Similarly, a tenfold variation for error rates in INDEL has been reported in a previous cross-platform comparison [19]. Low concordance may result from the different PCR steps used in library construction protocols [19] or could be interfered by plant RNA editing processes. Furthermore, as a means of intra-platform comparison, both strand-specific (BGISEQ-500 PE100) and strand-non-specific libraries (BGISEQ-500 PE75) have been applied for comparison in transcriptome analysis. From our results, no apparent differences were detected between the above two types of libraries. The only variation observed between these two approaches was in the identification of certain AS events, suggesting that the application of strand-specific library could achieve similar results to that of non-strand-specific library in BGISEQ-500 platform.

It is obvious that the biological significance is crucial for omics-based analysis, where the outcome which can be achieved from the experimental design largely relies on the consistency of sequence data. Yet, the considerably large variations observed among different sequencing platforms may lead to false interpretations in transcriptome studies. Previous publications suggested that variations in RNA sequencing can be easily avoided by standardizing sequencing protocols, platforms and bioinformatic analytical pipelines for specific experiment [19]. However, this could be effective for studies which rely on a single type of platform, but not for large-scale comparative studies, where the analysis has to be dealt with discrete data generated from different platforms. Therefore, approaches which ensure increased inter-platform consistency, differentiate real events from platform-specific bias and define the standard to manipulate the cross-platform data variations need to be extensively discussed within research community. Conducting cross-platform comparisons may help us further understand the signature of platform-specific variation. Furthermore, deepening the sequencing depth may increase the possibility to identify low abundance transcripts and splicing junctions [19], while nullifying possible inadequacies of the sequencing method. However, how this dynamic range is related to platform specific variation requires further investigation.

### Potential applications in cross-platform comparison

Cross-platform comparative studies provide valuable and indispensable information on sample repeatability and reproducibility, platform preference, bias estimation and the potential application of new sequencing technologies. The datasets generated from cross-platform comparisons are necessary for platform improvement, bias correction and development of suitable analytical tools for omics-based approaches. In the field of transcriptome, this will benefit for parameters like gene detection and quantification, DEG and AS identification, SNP and INDEL observation. In addition to standard analysis, other valuable information of transcriptome features such as natural antisense transcript detection, gene fusions, post-transcriptional modifications (*e.g.* RNA editing) could be retrieved by cross-platform studies, and even degraded samples could be addressed [19, 32], providing pivotal information for genome annotation refinement and mechanistic studies. Given the fast advancement in NGS sequencing platform, the practical multi-platform evaluation and development of standard protocols needs to keep pace.

## Conclusion

From the reference dataset generated by BGISEQ-500, we compared basic parameters in transcriptome analysis between this new sequencing platform and Illumina HiSeq4000, elucidating the capability of BGISEQ-500 as an alternative choice and yet another competent platform for plant transcriptome analysis. This case study may encourage more attempts to test their transcriptomic data using BGISEQ-500. We look forward to further explore the potential of this sequencing platform using a wider range of samples.

## Methods

### Plant material, growth conditions and abscisic acid treatment

In general, *Arabidopsis thaliana* WT seeds (Col-0 background) were surface-sterilized with 20% bleach for 30 min followed by four washes with distilled water. Subsequently, sterilized seeds were sown on Murashige and Skoog (MS) plates [33] supplemented with 0.8–1.0% (w/v) agar and 1.5% (w/v) sucrose. Plates were then incubated under 16 h light (23 °C)/8 h dark (21 °C) cycles following 2 days stratification. Twelve-day-old seedlings were treated with DMSO control or 50 μM ABA for 6 h and seedlings were harvested and subjected to further transcriptomic analysis.

### Plant RNA extraction

Total RNA of *Arabidopsis* seedlings was extracted using the RNeasy Mini Kit (Qiagen, Germany) according to the manufacturer’s bench protocol. Two samples, DMSO- and ABA-treated seedlings, each with three biological replicates were subjected to RNA extraction. The extracted RNA was then quantified and assessed for integrity using the NanoDrop (Thermo, USA) and 2100 Agilent Bioanalyzer (Agilent, USA) prior to subsequent experiments.

### Library construction and RNA sequencing in BGISEQ-500 platform

The strand non-specific library construction of PE75 mode was described as follows. In total, approximately 1 μg total RNA was initially used for BGISEQ-500 library construction. In general, DNase I was initially used to degrade double-stranded and single-stranded DNA contaminant in RNA samples. The mRNA molecules were then purified from total RNA using oligo(dT)-attached magnetic beads and fragmented into small pieces. First-strand cDNA was generated using random hexamer-primed reverse transcription, followed by a second-strand cDNA synthesis. The cDNA thus synthesized was subjected to end-repair and 3′ adenylation. Subsequently, adaptors were ligated to the ends of these 3′ adenylated cDNA fragments. The double stranded PCR products were heat denatured and circularized by the splint oligo sequence. The single stranded circular DNAs were formatted as the final library for Agilent Technologies 2100 bioanalyzer validation and subsequent PE75 sequencing.

For PE100 strand-specific library preparation, the first step in the workflow involved purifying the poly-A containing mRNA molecules using poly-T oligo-attached magnetic beads. Following purification, the mRNA was fragmented into small pieces using divalent cations under elevated temperature. The cleaved RNA fragments were copied into first strand cDNA using reverse transcriptase and random primers. This was followed by second strand cDNA synthesis using DNA Polymerase I and RNase H. This process removes the RNA template and synthesizes a replacement strand, incorporating dUTP in place of dTTP to generate dscDNA. The incorporation of dUTP quenched the second strand during amplification. These cDNA fragments were added with a single ‘A’ base and subsequently ligated to the adapter. The resultant product was purified and enriched with PCR amplification to yield the final cDNA library. The PCR yield was quantified and was subjected to single strand circularized DNA molecule (ssDNA circle) preparation for final library construction. DNA nanoballs (DNBs) were generated with the ssDNA circle by rolling circle replication (RCR) to intensify the fluorescent signals during the sequencing process. The DNBs were then loaded into the patterned nanoarrays and pair-end reads of 100 bp were read through on the BGISEQ-500 platform for subsequent data analysis.

### Library construction and RNA sequencing in Illumina HiSeq4000 platform

The library construction in HiSeq series was carried out according to the bench manual of TruSeq RNA Sample Prep Kit v2 (Illumina). Briefly, approximately 1 µg of total RNA sample was purified using oligo-dT beads, followed by fragmentation with Elute, Prime, Fragment Mix. First-strand cDNA was generated by First Strand Master Mix and Super Script II (Invitrogen) reverse transcription (Reaction condition:25 °C for 10 min, 42 °C for 50 min, 70 °C for 15 min). The product was purified (Agencourt RNAClean XP Beads, Agencourt), prior to the addition of Second Strand Master Mix and dATP, dGTP, dCTP, dUTP mix, to proceed with the synthesis of second-strand cDNA (16 °C for 1 h). The purified fragmented cDNA was incubated at 30 °C for 30 min in presence of End Repair Mix. Subsequently, the end-repaired cDNA was purified with Ampure XP Beads (Agencourt). A-Tailing Mix was then added, mixed and incubated at 37 °C for 30 min. The 3′end adenylated cDNA, RNA index adapter and ligation mix were combined and mixed, then incubated at 30 °C for 10 min. The end-repaired cDNA thus produced was then purified with Ampure XP Beads (Agencourt). The uracil-N-glycosylase enzyme was added into the reaction mixture at the final purification step, incubated at 37 °C for 10 min and the resulting product was purified using Agencourt Ampure XP Beads. Several rounds of PCR amplification with PCR primer cocktail and PCR master mix were performed to enrich the cDNA fragments, prior to the purification of PCR products with Ampure XP Beads (Agencourt). The library quality was assessed by checking the distribution of the fragments size using the Agilent 2100 bioanalyzer (Agilent DNA 1000 Reagents), and the library was quantified by using qRT-PCR (TaqMan Probe). The resultant library was subjected to Illumina HiSeq sequencing.

### Cross-platform RNA-sequencing data analysis

The bioinformatic pipeline was performed as described previously with minor modifications [27, 28]. Raw sequencing reads were filtered to get clean reads by using SOAPnuke (v1.5.2, parameters -l 15, -q 0.2, -n 0.05) (https://github.com/BGI-flexlab/SOAPnuke). For both BGISEQ-500 and HiSeq4000 derived sequencing data, HISAT pipeline [34] was applied to align reads against reference genome. StringTie [35] was then used for transcript reconstruction. Subsequently, Cuffcompare (Cufflinks tools) [36] was utilized to compare reconstructed transcripts and the reference annotation of *Arabidopsis*. Coding potential of novel transcripts were predicted by CPC [37]. SNP and INDEL calling was carried out by using GATK (v 3.4-0, https://www.broadinstitute.org/gatk) [38] with parameters (call): -allowPotentiallyMisencodedQuals, -stand_call_conf 20.0, -stand_emit_conf 20.0 and parameters (filter): -window 35, -cluster 3, -filterName FS, -filter “FS > 30.0”, -filterName QD, -filter “QD < 2.0”. In addition, we have mapped clean reads to reference genes using Bowtie2 software [39]. Expression values of candidate genes were then calculated by RSEM [40]. The identification of DEGs was based on the negative binomial distribution of DEseq2 package [41], AudicS [42], Cuffdiff [43], DEGseq [44] and edgeR [45]. The cutoff of DEGs was Fold Change ≥ 2 and adjusted *P* value ≤ 0.05. The subsequent GO and pathway analysis was followed by previous description [21, 46–48]. Alternative spliced events were identified according to previous description by using ASprofile software (http://ccb.jhu.edu/software/ASprofile) [49]. In brief, as described previously [21], AS junctions supported with two or more reads were subsequently used for AS events identifications.

## Additional files

**Additional file 1: Figure S1.** Schematic view of library construction procedures of BGISEQ-500 in this study. **Figure S2.** Base composition among three sequencing approaches. **Figure S3.** Comparison of method-specific gene quantification. **Figure S4.** Repeatability of gene quantification. **Figure S5.** GO analysis of DEGs identified by three sequencing approaches. **Figure S6.** Methods in DEGs identification and comparisons of biological interpretation. **Figure S7.** Pathway enrichment of each sequencing approach by using DEG calling software AudicS. **Figure S8.** Pathway enrichment of each sequencing approach by using DEG calling software Cuffdiff. **Figure S9.** Pathway enrichment of each sequencing approach by using DEG calling software DEGseq. **Figure S10.** Pathway enrichment of each sequencing approach by using DEG calling software edgeR. **Figure S11.** Inter-platform comparison for AS events identification. **Figure S12.** Intra-platform comparison of AS identification by BGISEQ-500 PE75 approach. **Figure S13.** Intra-platform comparison of AS identification by BGISEQ-500 PE100 approach. **Figure S14.** Intra-platform comparison of AS identification by HiSeq4000 PE100 approach. **Figure S15.** Intra-platform comparison of AS identification by three approaches. **Figure S16.** Intra- and inter-platform comparison for SNP identification. **Figure S17.** Intra- and inter-platform comparison for INDEL identification.

**Additional file 2: Table S1.** Summary of gene and transcript identification.
