# Supplementary material for: Comparative performance of the BGISEQ-500 and Illumina HiSeq4000 sequencing platforms for transcriptome analysis in plants
Source: Plant Methods. 2018 Aug 13;14:69. doi: 10.1186/s13007-018-0337-0 (PMC6088413; doi:10.1186/s13007-018-0337-0)
Supplement: Supplementary file 1 — Additional file 1: Figure S1. Schematic view of library construction procedures of BGISEQ-500 in this study. Figure S2. Base composition among three sequencing approaches. Figure S3. Comparison of method-specific gene quantification. Figure S4. Repeatability of gene quantification. Figure S5. GO analysis of DEGs identified by three sequencing approaches. Figure S6. Methods in DEGs identification and comparisons of biological interpretation. Figure S7. Pathway enrichment of each sequencing approach by using DEG calling software AudicS. Figure S8. Pathway enrichment of each sequencing approach by using DEG calling software Cuffdiff. Figure S9. Pathway enrichment of each sequencing approach by using DEG calling software DEGseq. Figure S10. Pathway enrichment of each sequencing approach by using DEG calling software edgeR. Figure S11. Inter-platform comparison for AS events identification. Figure S12. Intra-platform comparison of AS identification by BGISEQ-500 PE75 approach. Figure S13. Intra-platform comparison of AS identification by BGISEQ-500 PE100 approach. Figure S14. Intra-platform comparison of AS identification by HiSeq4000 PE100 approach. Figure S15. Intra-platform comparison of AS identification by three approaches. Figure S16. Intra- and inter-platform comparison for SNP identification. Figure S17. Intra- and inter-platform comparison for INDEL identification. [file 13007_2018_337_MOESM1_ESM.docx]

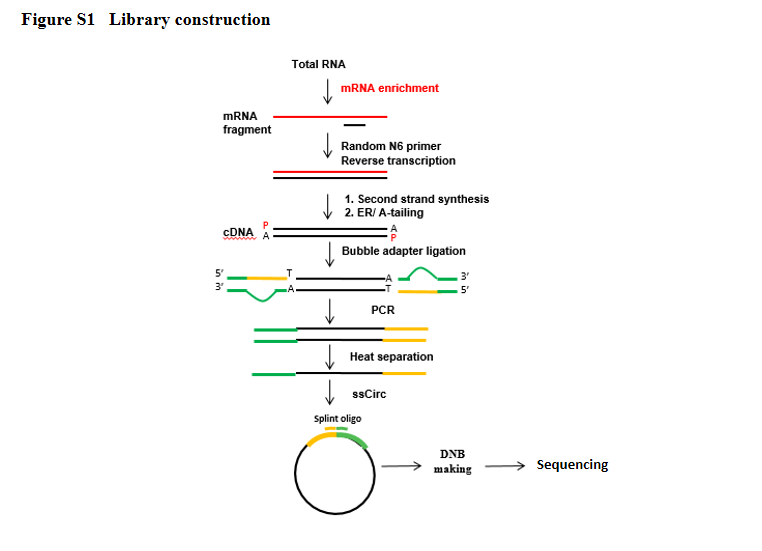


**Figure S1 Schematic view of library construction procedures of BGISEQ-500 in this study.**

Abbreviations: mRNA, messenger RNA; ER/A tailing, enzyme mix for adapter ligation; PCR, polymerase chain reaction; ssCirc, single strand circle DNA; DNB, DNA nanoballs.


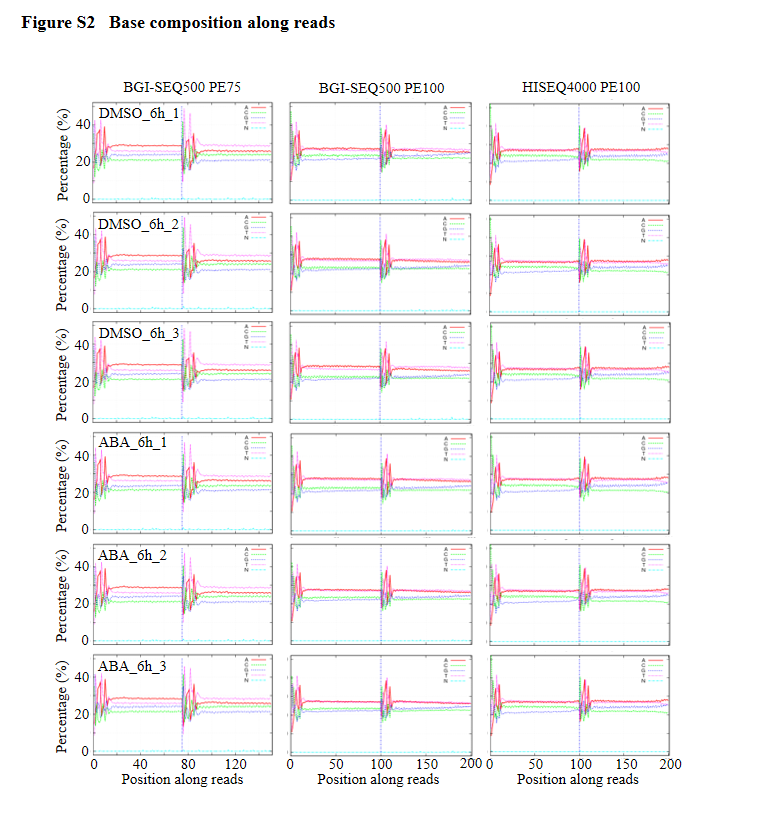


**Figure S2** **Base composition among three sequencing approaches.** The percentage of base composition is graphed against position along reads.


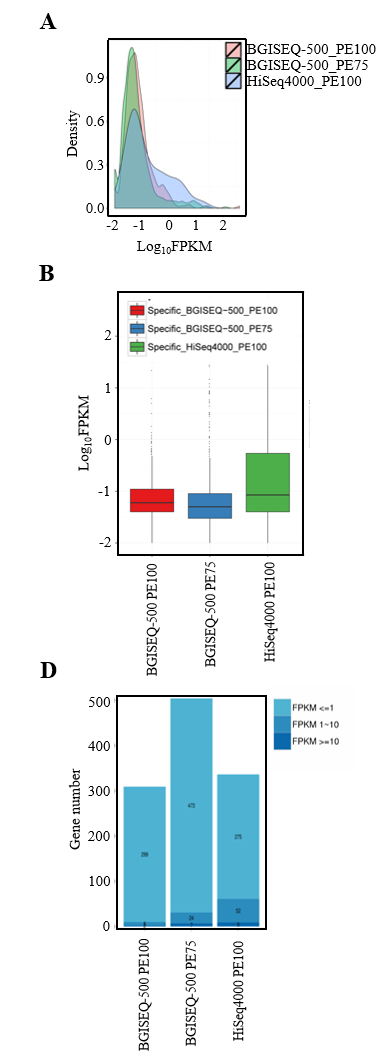


**Figure S3 Comparison of method-specific gene quantification.** Expression density distribution (A), boxplot gene expression graph (B), High and low abundance transcripts quantification (C) for all the replicates tested by three sequencing approaches in this study.
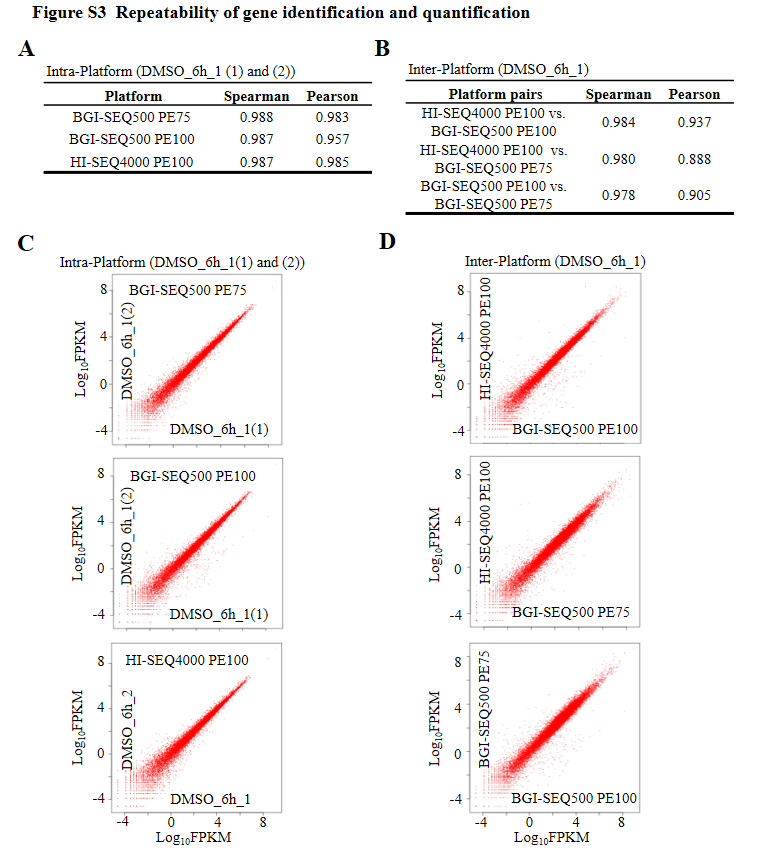


**Figure S4** **Repeatability of gene quantification.** Intra-platform (A, C) and inter-platform (B, D) comparison of gene quantification among three sequencing approaches.


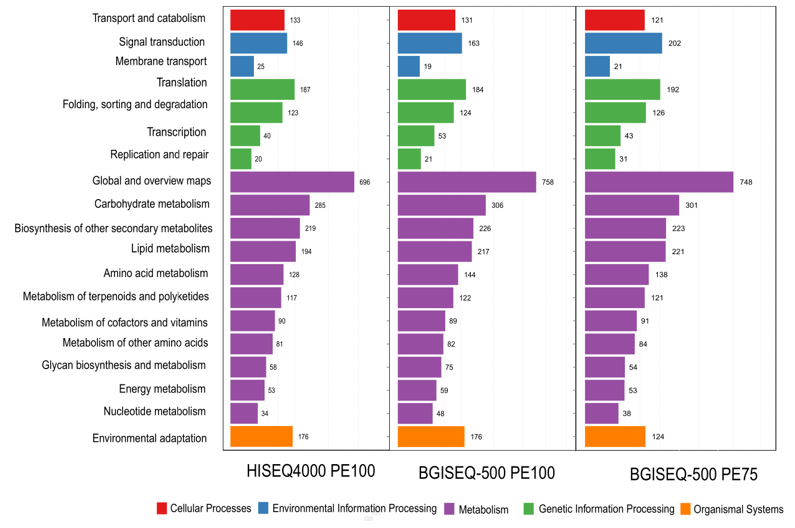


**Figure S5 GO analysis of DEGs identified by three sequencing approaches.** Functional classification and summary of DEGs identified by DEseq2 method.


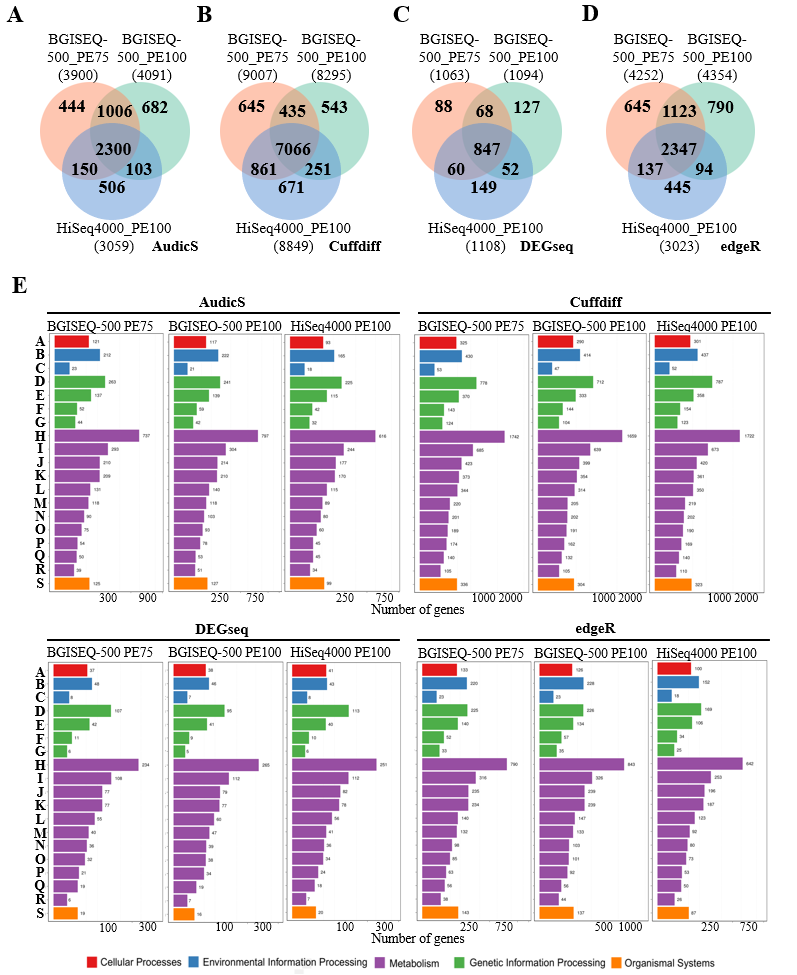


**Figure S6 Methods in DEGs identification and comparisons of biological interpretation.** (A-D) Venn diagrams represent DEGs identified by four different methods. (E) Functional summary of DEGs identified by four different approaches. A, Transport and catabolism; B, Signal transduction; C, Membrane transport; D, Translation; E, Folding, sorting and degradation; F, Transcription; G, Replication and repair; H, Global and overview maps; I, Carbohydrate metabolism; J, Biosynthesis of other secondary metabolites; K, Lipid metabolism; L, Amino acid metabolism; M, Metabolism of terpenoids; N, Metabolism of cofactors and vitamins; O, Metabolism of other amino acids; P, Energy metabolism; Q, Glycan biosynthesis and metabolism; R, Nucleotide metabolism; S, Environmental adaptation.


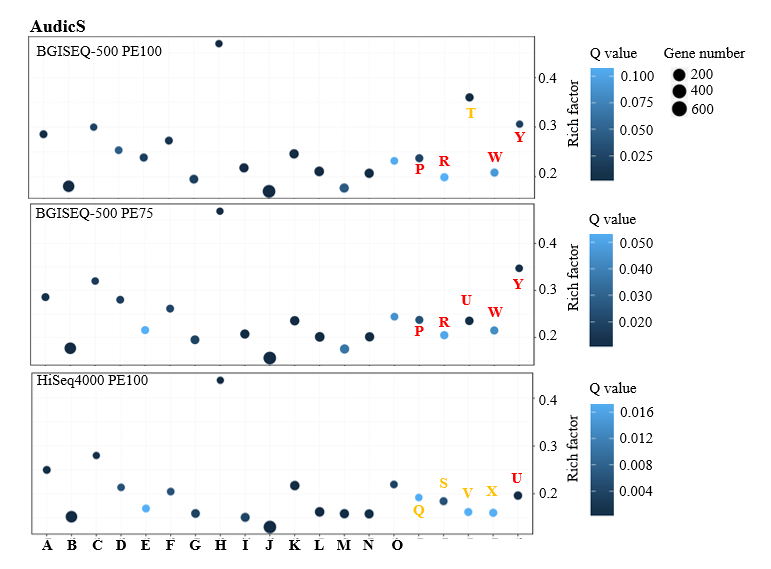


**Figure S7 Pathway enrichment of each sequencing approach by using DEG calling software AudicS.** Black, pathways enriched in all the three approaches; Red, pathways enriched in two approaches; Orange, pathways enriched in one approach. A, alpha-Linolenic acid metabolism; B, Biosynthesis of secondary metabolites; C, Biosynthesis of unsaturated fatty acids; D, Carotenoid biosynthesis; E, Cutin, suberine and wax biosynthesis; F, Flavonoid biosynthesis; G, Glycerolipid metabolism; H, Indole alkaloid biosynthesis; I, MAPK signaling pathway; J, Metabolic pathways; K, Phenylpropanoid biosynthesis; L, Plant hormone signal transduction; M, Ribosome; N, Starch and sucrose metabolism; O, Other terpenoid biosynthesis; P, Fatty acid metabolism; Q, Ether lipid metabolism; R, Peroxisome; S, Galactose metabolism; T, Photosynthesis; U, Ribosome biogenesis in eukaryotes; V, Other glycan degradation; W, Biosynthesis of secondary metabolites in phenylpropanoid pathway; X, Porphyrin and chlorophyll metabolism; Y, Zeatin biosynthesis.


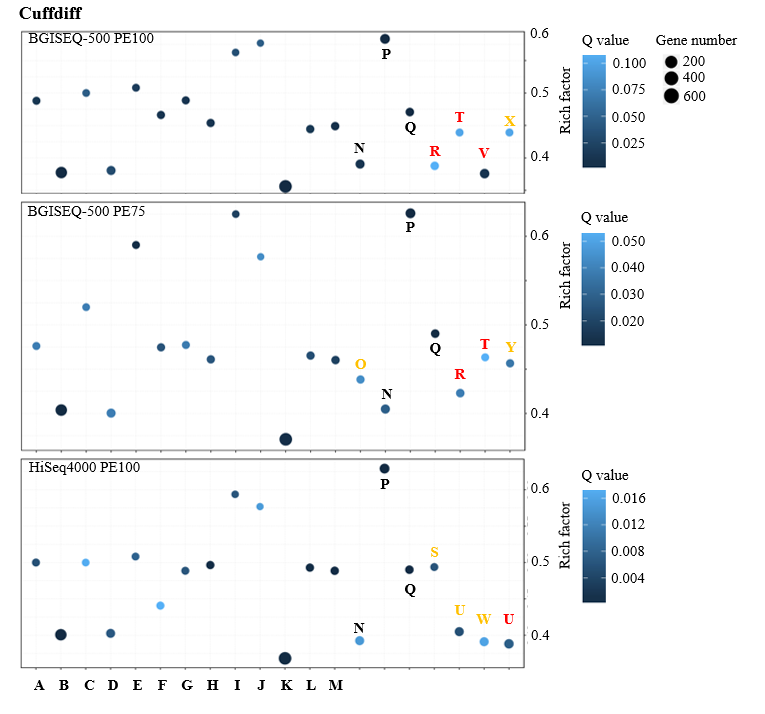


**Figure S8 Pathway enrichment of each sequencing approach by using DEG calling software Cuffdiff.** Black, pathways enriched in all the three approaches; Red, pathways enriched in two approaches; Orange, pathways enriched in one approach. A, alpha-Linolenic acid metabolism; B, Biosynthesis of secondary metabolites; C, Biosynthesis of unsaturated fatty acids; D, Carbon metabolism; E, Fatty acid elongation; F, Fatty acid metabolism; G, Flavonoid biosynthesis; H, Galactose metabolism; I, Indole alkaloid biosynthesis; J, Isoflavonoid biosynthesis; K, Metabolic pathways; L, Pentose phosphate pathway; M, Peroxisome; N, Phenylpropanoid biosynthesis; O, Phagosome; P, Ribosome; Q, Ribosome biogenesis in eukaryotes; R, Circadian rhythm; S, Alanine, aspartate, glutamate metabolism; T, Citrate cycle; U, Biosynthesis of amino acids; W, MAPK signaling pathway; X, Other terpenoid biosynthesis; Y, Glutathione metabolism.


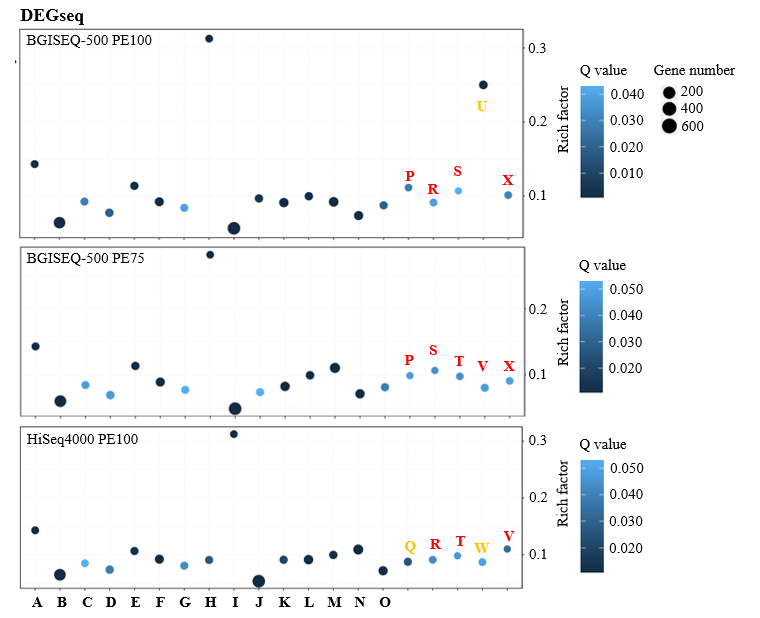


**Figure S9 Pathway enrichment of each sequencing approach by using DEG calling software DEGseq.** Black, pathways enriched in all the three approaches; Red, pathways enriched in two approaches; Orange, pathways enriched in one approach. A, alpha-Linolenic acid metabolism; B, Biosynthesis of secondary metabolites; C, Cutin, suberine and wax biosynthesis; D, Cyanoamino acid metabolism; E, Galactose metabolism; F, Glycerolipid metabolism; G, Glyoxylate and dicarboxylate metabolism; H, Indole alkaloid biosynthesis; I, Metabolic pathways; J, Peroxisome; K, Phenylpropanoid biosynthesis; L, Porphyrin and chlorophyll metabolism; M, Ribosome; N, Starch and sucrose metabolism; O, Terpenoid backbone biosynthesis; P, Alanine, aspartate, glutamate metabolism; Q, Glycine, serine and threonine metabolism; R, Arginine and proline metabolism; S, Carotenoid biosynthesis; T, Fatty acid degradation; U, Photosynthesis; V, Biosynthesis of secondary metabolites in phenylpropanoid pathway; W, Glutathione metabolism; X, Tyrosine metabolism.


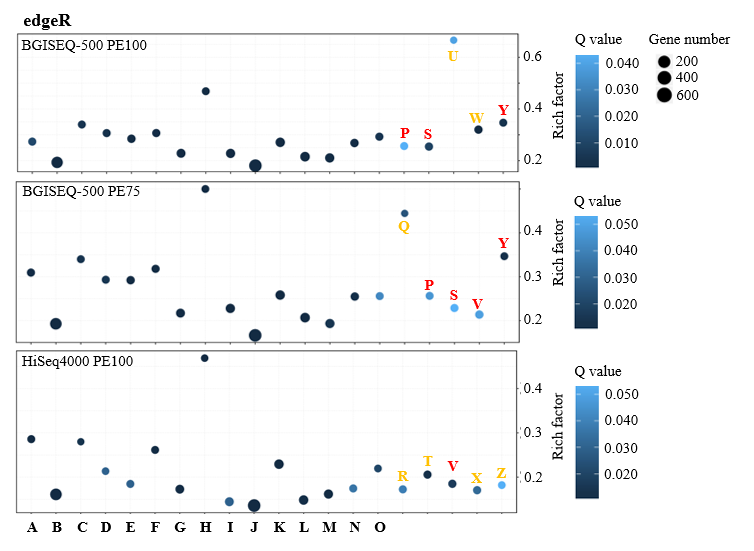


**Figure S10 Pathway enrichment of each sequencing approach by using DEG calling software edgeR.** Black, pathways enriched in all the three approaches; Red, pathways enriched in two approaches; Orange, pathways enriched in one approach. A, alpha-Linolenic acid metabolism; B, Biosynthesis of secondary metabolites; C, Biosynthesis of unsaturated fatty acids; D, Carotenoid biosynthesis; E, Cutin, suberine and wax biosynthesis; F, Flavonoid biosynthesis; G, Glycerolipid metabolism; H, Indole alkaloid biosynthesis; I, MAPK signaling pathway; J, Metabolic pathway; K, Phenylpropanoid biosynthesis; L, Plant hormone signal transduction; M, Starch and sucrose metabolism; N, Biosynthesis of secondary metabolites in phenylpropanoid pathway;; O, Other terpenoid biosynthesis; P, Ether lipid metabolism; Q, Anthocyanin biosynthesis; R, Fructose and mannose metabolism; S, Fatty acid metabolism; T, Galactose metabolism; U, Glycosphingolipid biosynthesis; V, Photosynthesis; W, Other glycan degradation; X, Peroxisome; Y, Zeatin biosynthesis; Z, Tyrosine metabolism.


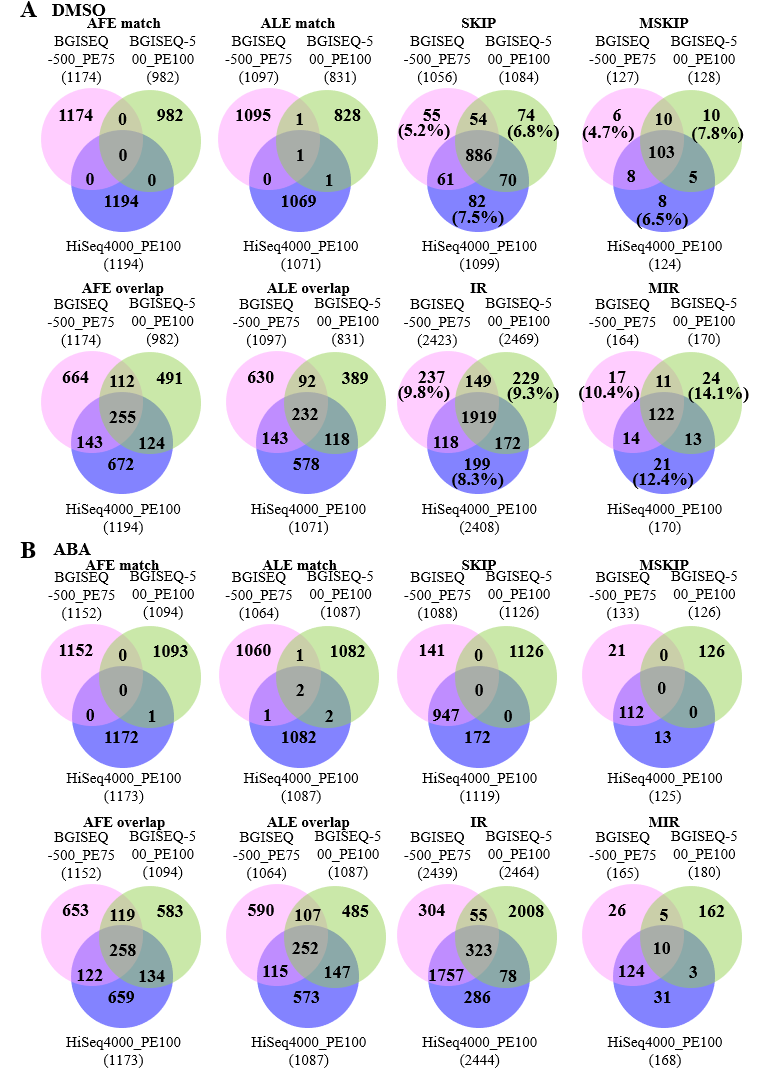


**Figure S11 Inter-platform comparison for AS events identification.** Venn diagrams to represent (A) AS events in DMSO-treated and (B) ABA-treated samples. AFE, alternative first exon; ALE, alternative last exon; IR, intron retention; MIR, multiple intron retention; SKIP, exon skipping; MSKIP, multiple exon skipping.


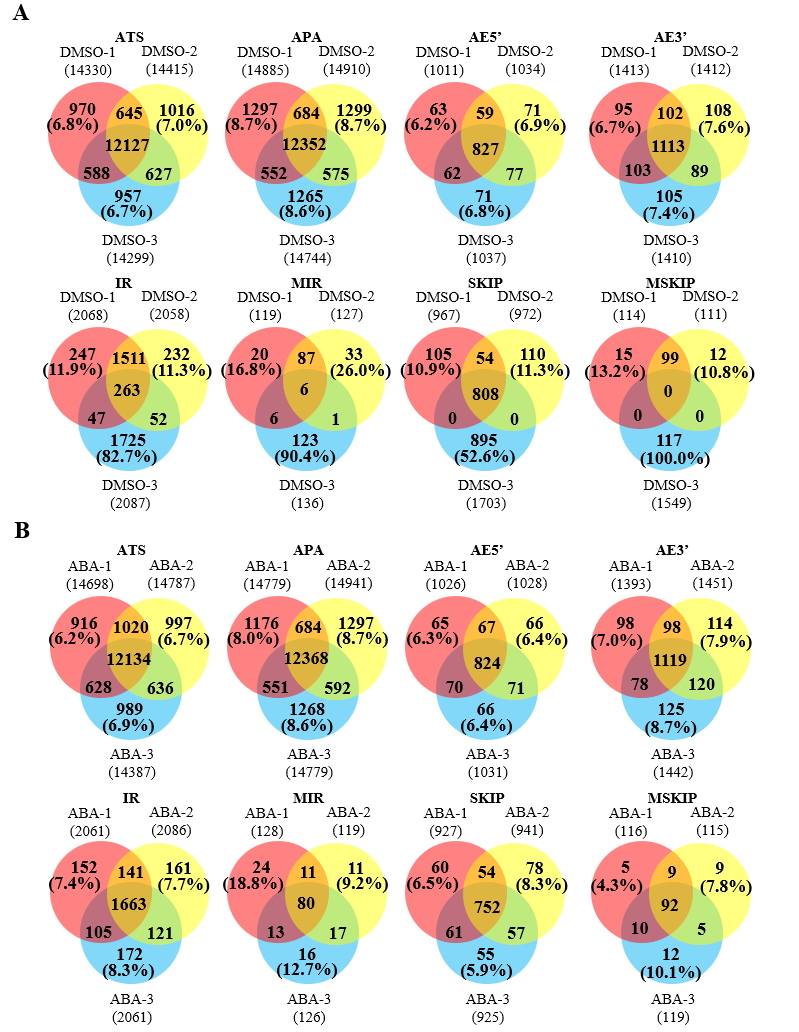


**Figure S12 Intra-platform comparison of AS identification by BGISEQ-500 PE75 approach.** Venn diagrams to represent (A) AS events in DMSO-treated and (B) ABA-treated samples. ATS, alternative transcription start; APA, alternative polyadenylation; AE5’, alternative 5’ splice site; AE3’, alternative 3’ splice site; IR, intron retention; MIR, multiple intron retention; SKIP, exon skipping; MSKIP, multiple exon skipping.


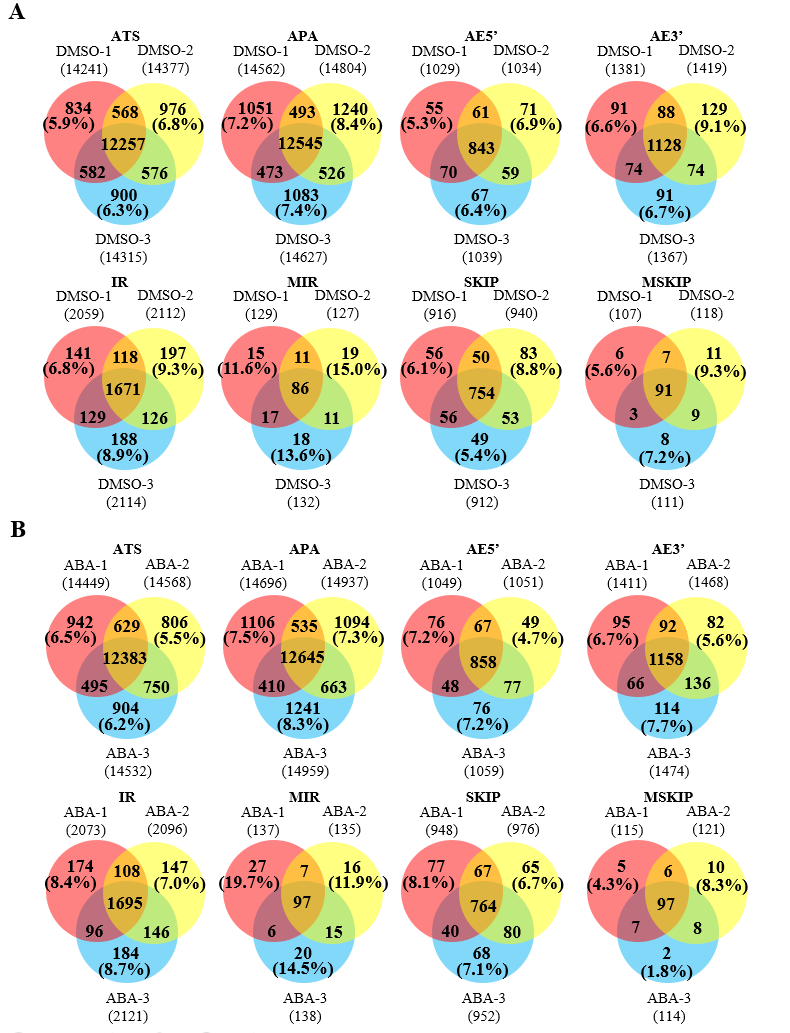


**Figure S13 Intra-platform comparison of AS identification by BGISEQ-500 PE100 approach.** Venn diagrams to represent (A) AS events in DMSO-treated and (B) ABA-treated samples. ATS, alternative transcription start; APA, alternative polyadenylation; AE5’, alternative 5’ splice site; AE3’, alternative 3’ splice site; IR, intron retention; MIR, multiple intron retention; SKIP, exon skipping; MSKIP, multiple exon skipping.


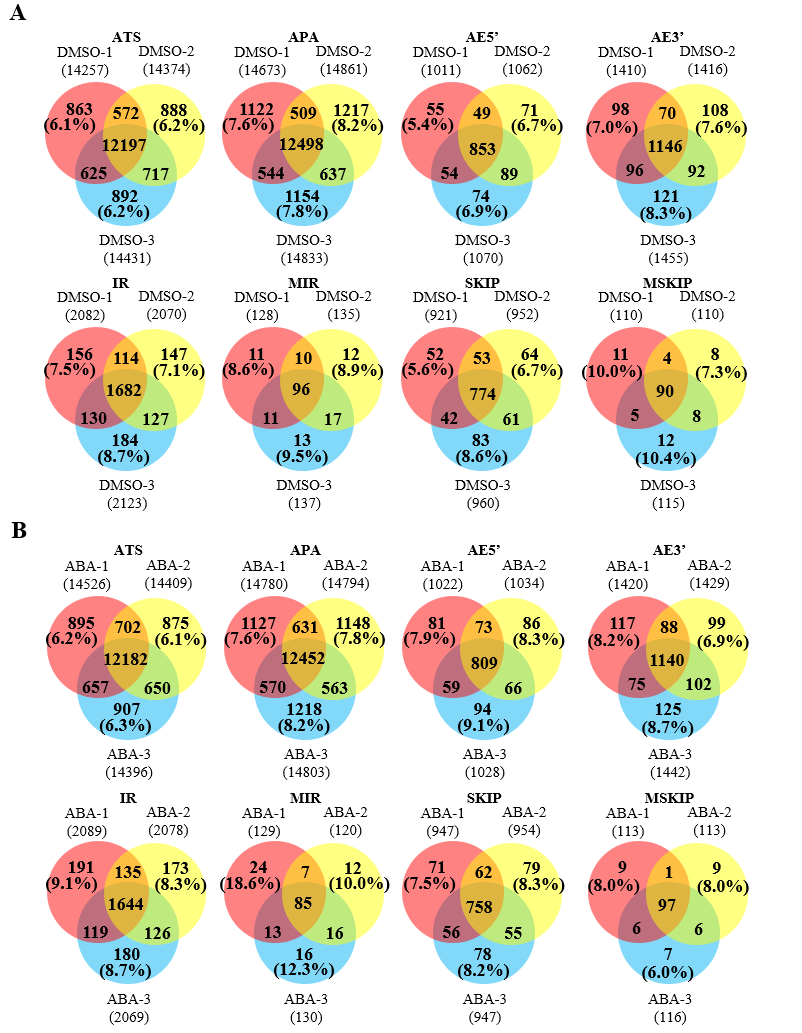


**Figure S14 Intra-platform comparison of AS identification by HiSeq4000 PE100 approach.** Venn diagrams to represent (A) AS events in DMSO-treated and (B) ABA-treated samples. ATS, alternative transcription start; APA, alternative polyadenylation; AE5’, alternative 5’ splice site; AE3’, alternative 3’ splice site; IR, intron retention; MIR, multiple intron retention; SKIP, exon skipping; MSKIP, multiple exon skipping.


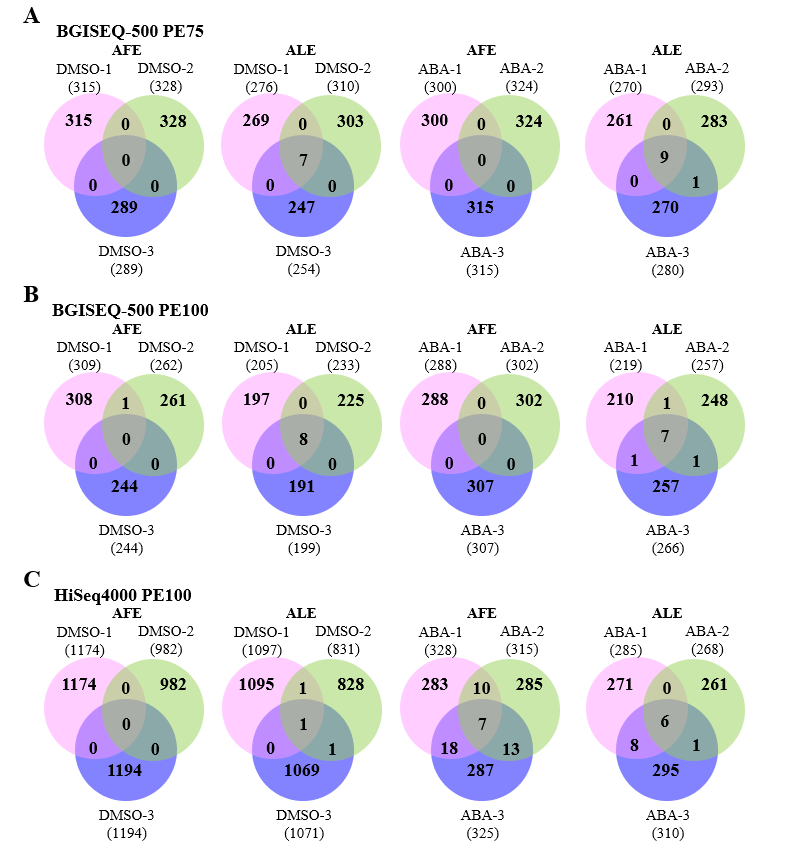


**Figure S15 Intra-platform comparison of AS identification by three approaches.** Venn diagrams to represent AS events in DMSO-treated and ABA-treated samples by using (A) BGISEQ-500 PE75, (B) BGISEQ-500 PE100 and (C) HiSeq4000 PE100 approaches. AFE, alternative first exon; ALE, alternative last exon.


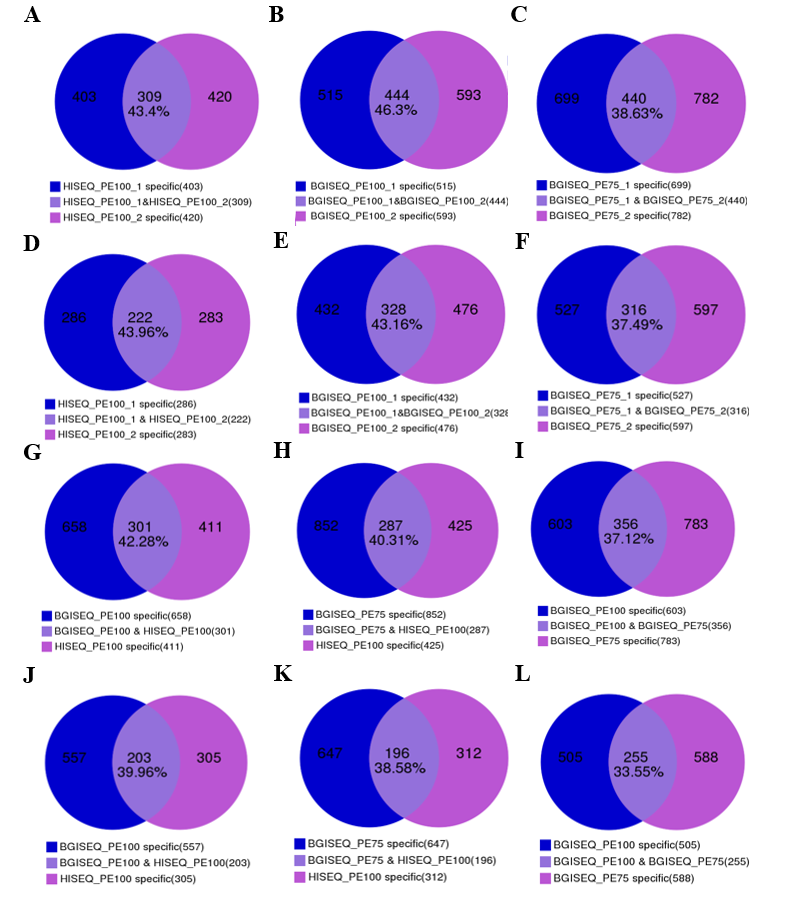


**Figure S16 Intra- and inter-platform comparison for SNP identification.** Intra-platform (A-F) and inter-platform (G-L) comparison of SNP calling among three sequencing approaches. Statistical analysis is based on transcripts (A-C, G-I) or CDS sequences (D-F, J-L).


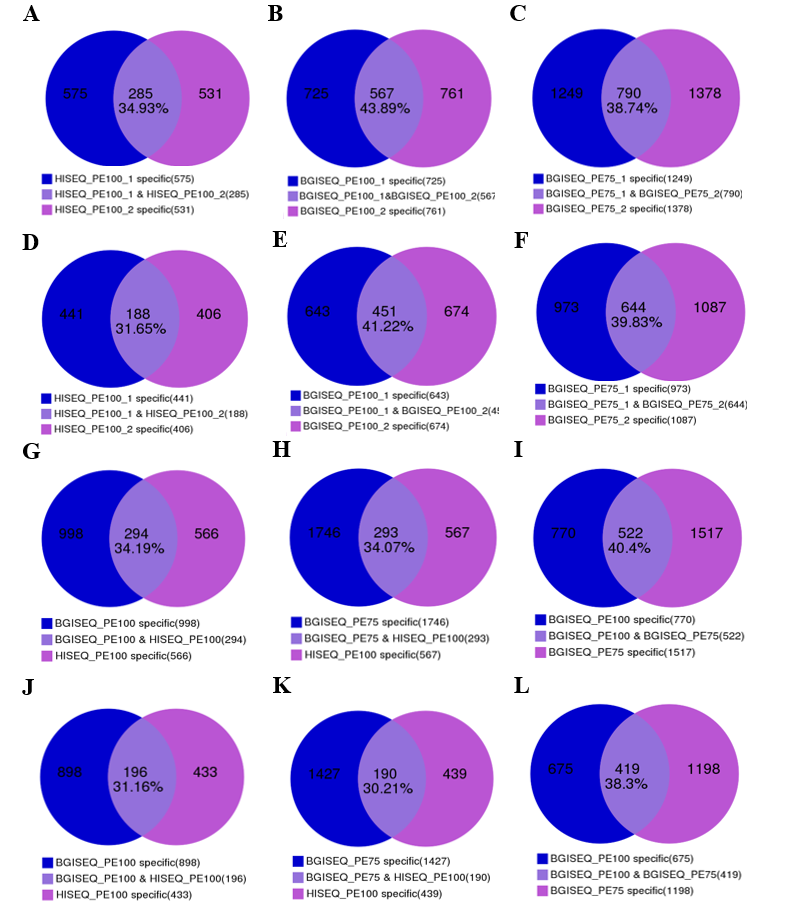


**Figure S17 Intra- and inter-platform comparison for INDEL identification.** Intra-platform (A-F) and inter-platform (G-L) comparison of INDEL calling among three sequencing approaches. Statistical analysis is based on transcripts (A-C, G-I) or CDS sequences (D-F, J-L).
