# Supplementary material for: Comparative performance of the BGISEQ-500 and Illumina HiSeq4000 sequencing platforms for transcriptome analysis in plants
Source: Plant Methods. 2018 Aug 13;14:69. doi: 10.1186/s13007-018-0337-0 (PMC6088413; doi:10.1186/s13007-018-0337-0)
Supplement: Supplementary file 2 — Additional file 2: Table S1. Summary of gene and transcript identification. [file 13007_2018_337_MOESM2_ESM.docx]

Table S1 Summary of gene and transcript identification
